# Tracing socioeconomic inequalities in children’s and adolescent’s mental health: longitudinal study findings from 2017 to 2024

**DOI:** 10.1186/s12889-026-26577-0

**Published:** 2026-02-17

**Authors:** Franziska Reiss, Michael Erhart, Anne Kaman, Janine Devine, Fionna Zoellner, Ulrike Ravens-Sieberer

**Affiliations:** 1 Department of Child and Adolescent Psychiatry, Psychotherapy, and Psychosomatics, Research Division “Child Public Health”, University Medical Center Hamburg-Eppendorf, Martinistrasse 52, Hamburg, 20246 Germany; 2 Alice Salomon University of Applied Science, Alice-Salomon-Platz 5, Berlin, 12627 Germany

**Keywords:** Inequality, Mental health, Children, Adolescents, Income, Socioeconomic status, COPSY

## Abstract

**Background:**

Socioeconomic inequalities in mental health are already evident in early life. During the COVID-19 pandemic, many families experienced additional stress. This study examines the development of mental health problems in children and adolescents in relation to their family’s socioeconomic status in times of global crises, taking into account other risk and resource factors.

**Methods:**

Data were used from the population-based BELLA study (pre-pandemic, *N* = 1,580) and from the longitudinal COPSY study (post-/pandemic, *N* = 1,586-1,701) with participants aged 7 to 22 years. Surveys were conducted before (BELLA T0, 2017), during (COPSY T1-T5, 2020–2022) and after the COVID-19 pandemic (COPSY T6-T7, 2023–2024) in Germany. Mental health problems (SDQ), socioeconomic status (parental education, net equivalent household income), and additional factors (parental psychopathology, social support, family cohesion) were examined. Prevalence in mental health problems, differences in mean values (SDQ) and panel regression analyses were calculated.

**Results:**

Over the entire survey period, children and adolescents with a low socioeconomic status were more affected by mental health problems than their peers with a high status (e.g., pre-pandemic: 6.2% vs. 14.6%; post-pandemic: 11.0% vs. 18.3%). During the pandemic, mental health problems increased significantly in all status groups. Health inequality remained stable and even narrowed towards the end of the pandemic, whereby mean values in youth mental health problems were nearly the same for high and low educated parents in autumn 2022. After the pandemic, mental health inequalities started to re-increase. This pattern is also visible, but less pronounced, for household income. Personal resources, family cohesion, and social support served as protective factors for youth mental health.

**Conclusion:**

Socioeconomic inequalities in the mental health of children and adolescents remain evident in times of global crisis. Over the course of the pandemic, these mental health inequalities have not increased and even equalized, but post-pandemic recovery effects are only visible for young people in higher socioeconomic status. However, there is still an ongoing need for targeted health promotion and prevention that strengthens personal and social resources especially, but not only, for socially disadvantaged children and families at both an individual and societal level.

**Supplementary Information:**

The online version contains supplementary material available at 10.1186/s12889-026-26577-0.

## Introduction

For decades, there has been a robust social gradient between socioeconomic background and mental health. Children and adolescents from families with a low social status are affected by mental health problems around 2–3 times more frequently than their peers, as international studies have pointed out [1–4]. Compared to children from high socioeconomic families, those from low socioeconomic families have also a lower health-related quality of life and their families incurred higher government healthcare costs from birth until adolescence [4].

With the onset of the COVID-19 pandemic in 2020, a global health crisis placed an additional burden on children’s and adolescents’ daily life. International reviews underscore that the pandemic had immediate but also long-lasting detrimental effects on children’s well-being and mental health [5, 6]. This is especially relevant as poor mental health in childhood has a huge impact on their entire life course, including effects on children’s educational abilities and the persistence of childhood mental health problems into adulthood [7, 8].

During the pandemic, social isolation, remote learning, disrupted routines, and heightened family stress contributed to increased levels of anxiety, depression, and behavioural problems among children and adolescents [9]. This disruption affects a critical developmental period, though comprehensive long-term effects remain difficult to examine [10].

Thereby, this pandemic crisis seems to have especially affected those who were already at a higher risk for mental health problems [11–14]. For instance, recent research from France and the UK showed that families with lower socioeconomic status (SES; e.g., low-income households) were disproportionately affected by the pandemic [15, 16]. This social gradient is defined as an inverse linear relationship between family’s socioeconomic status and children’s mental health status, i.e. health is progressively better the higher the socioeconomic position of people and communities. Thereby, individual-level socioeconomic status of youth is predominantly measured by parental education and the average household income of the family [17]. Hence, although SES indicators may overlap, it is worth to investigate them as separate variables to better understand their unique effects on mental health [18]. For instance, a longitudinal study from France showed that a deterioration in the family’s financial situation during the pandemic led to an increase in ADHD and emotional symptoms in children [11], while low parental education was associated with higher mental health problems [19]. A systematic review including more than 300 articles of both cross-sectional/repeated cross-sectional and longitudinal studies highlighted the relationship between socioeconomic conditions and symptoms of emotional disorders during COVID-19 in a range from high- to low-income countries. Results showed that particularly economic concerns and financial strain predict depressive and anxiety symptoms in the adult population the best [20].

Nonetheless, only a few longitudinal studies have investigated the development of mental health inequalities of youths related to socioeconomic status during and after the COVID-19 pandemic. For instance, a representative UK Household Longitudinal Study found deteriorated mental health among 5- and 8-year-olds during the pandemic, although several inequalities narrowed [19]. Thereby, children from traditionally advantaged groups saw larger declines than children from disadvantaged groups, that is, child mental health has become more equal but at a worse overall level.

In these turbulent times of global crisis, a better understanding of the long-term trajectories and consequences of the pandemic for child mental health, and how it intersects with different domains of socioeconomic disadvantage, but also potential resilience, is essential for planning mental health service delivery and intervening on the causes of declining child mental health [19]. The pathways through which socioeconomic conditions influence children’s mental health are complex and inter-related, and do not only refer to individual/familial circumstances, but also to the distribution of power and resources that determine the economic, material, and psychosocial conditions in which children grow up [21]. Various social determinants of children’s and adolescents’ mental health play hereby an important role. In a Canadian qualitative study, for instance, pandemic-related public health measures and restrictions particularly affected the social determinants of education, access to health services, employment and income security, and social support amongst adolescents as online schooling, loss of connection with peers, income instability, and limited health services affected their mental health [22]. In our study, we will integrate different subdomains in the social determinants of health [3, 23]. The WHO’s Commission on the Social Determinants of Health defines those social determinants as “the conditions in which people are born, grow, live, work and age” (World Health Organization, 2008). In line with a recent review of subjective well-being [24], we conceptualise categories of risk factors and resources. Therefore, we will use the theoretical approach of social determinants of health [25]. All determinants were assessed in the longitudinal studies and include (a) individual determinants, such as gender, age, migration background, socioeconomic status, and parental psychopathology; (b) intermediary determinants, such as social support, family cohesion and personal resources; and (c) societal and structural determinants, such as the COVID-19 pandemic and the associated restrictions as well as other global crises to illustrate the main determinants of mental health in young people.

Despite a number of studies investigating mental health inequality in youth [1, 26], there is lack of longitudinal studies on children’s and adolescents’ mental health in the context of global crisis, taking into account various social determinants.

Therefore, our study aims to close this research gap by examining the association between socioeconomic status of the family (i.e., parental education and household income) and mental health problems in children and adolescents aged 7 to 22 years in Germany, covering a time span from before to after the COVID-19 pandemic. In addition, several potential personal and social risk and resource factors of mental health in children and adolescents are explored.

The present study investigates the following research questions:

1. How do children and adolescents differ in their mental health according to their socioeconomic status before, during, and after the COVID-19-pandemic in Germany?
2. Are mental health inequalities in children and adolescents increasing in turbulent times, i.e. the time from pre- until post-COVID-19 pandemic in Germany?
3. Which risk or resource factors (e.g., income, parental education, parental psychopathology, personal resources, family cohesion, social support) are associated with children’s and adolescents’ mental health?

Concerning research question 1, it is hypothesized that low parental SES is associated with higher mental health problems in children and adolescence before, during and after the COVID-19-pandemic. In terms of research question 2, it is hypothesized that mental health inequalities between children with a low SES (i.e. low parental education, low household income) compared to children with a high SES (i.e. high parental education, high household income) increased during the COVID-19 pandemic; and with reference to research question 3, it is hypothesized that other risk or resource factors (e.g. parental psychopathology, personal resources, family cohesion, social support) are associated with children’s and adolescents’ mental health.

## Method

### Study design and participants

Pre-pandemic mental health data for children and adolescents were obtained from the population-based study ‘Behaviour and Well-being of Children and Adolescents in Germany’ (BELLA). BELLA is a module focused on mental health and health-related quality of life within the ‘German Health Interview and Examination Survey for Children and Adolescents’ (KiGGS), conducted by the Robert Koch-Institute, the central institution of the German Federal Government responsible for disease surveillance and prevention. The BELLA study was conducted between 2014 and 2017 and included n = 1,580 participants (aged 11 to 19 years). All participants and their parents were informed about the study procedures, data protection and voluntary participation. Informed consent was gathered from the parents of children under the age of 18 years and from adolescents aged at least 14 years. Data assessment was conducted by an age- and target-specific questionnaire. The BELLA study was approved by the Federal Commissioner for Data Protection and received a positive vote from the Ethics Committee of Hamburg’s Chamber of Psychotherapists (on 24 September 2014). For further information on study design and methodology see Otto, Reiss [27].

Pandemic and post-pandemic data was taken from the population-based ‘COvid-19 and PSYchological Health’ study (COPSY), which is one of the first large-scale studies that monitored mental health in children and adolescents during and after the COVID-19 pandemic in Germany. The survey is based on the study design of the BELLA study. The COPSY study was conducted in seven survey waves: T1: May–June 2020, T2: December 2020–January 2021, T3: September–October 2021, T4: February 2022, T5: September–October 2022, T6: October-November 2023, and T7: October 2024. In each survey wave, between 1,586 (T1) and 1,505 (T7) families took part in the survey. Families who already participated were re-invited to participate in follow-up surveys, while new families were recruited to compensate for drop-outs to ensure socio-demographic representativeness and comparability between the survey waves. The initial participation rate was 45.8% and the participants took part in an average of 56.7% of the survey waves (T2-T7). Families were invited via email to participate in a nationwide survey, which used quota sampling. This method helped to ensure the sample reflected the sociodemographic characteristics of the German population. The data was adjusted using weights from the most recent microcensus to ensure that the sample accurately reflects the main sociodemographic characteristics of the German population. Written informed consent to participate in this study was provided by the participants’ legal guardian. The COPSY study was approved by the Local Psychological Ethics Committee (LPEK-0151) and the Commissioner for Data Protection of the University of Hamburg. Further information on the design have been described elsewhere [28–30].

### Measurements

#### Sociodemographic

Sociodemographic variables covered questions on the age and gender of children and adolescents at baseline. The gender categories included “female”, “male”, and for the COPSY study also “diverse”. Additionally, we assessed information on migration background by two questions and single parenthood by one question.

#### Socioeconomic status

*Parental education* (reported by the parents) as an indicator of family socioeconomic status was assessed by the Comparative Analyses of Social Mobility in Industrial Nations (CASMIN classification of education [31]. This distinguishes between nine educational groups based on the combination of school and vocational qualifications. Parents were classified into “low *education”* (e.g., incomplete education, general elementary school, or basic vocational training) versus “medium/high education” (e.g., intermediate secondary diplomas, Abitur or A-Level, degrees from universities or technical colleges). In standard two-parent households, the highest education level attained by either parent was used to represent the parental education. In incomplete families (e.g., divorced or single parents) the education level of the responding parent (usually the mother/custodial parent) was used as the sole measure.

*Financial situation* (reported by the parents) was assessed by the needs-weighted net household income (net equivalent income), which is in line with the requirements of the German Federal Government’s poverty and wealth report. The net equivalent income *(household income)* is a net income weighted according to the size of the household and age of its members. According to the modified scale generally used by the Organization for Economic Cooperation and Development (OECD-modified equivalence scale), the main income earner of the household receives a weighting factor of 1.0, all other household members aged 14 and over a factor of 0.5 and persons under 14 a factor of 0.3 [32]. Income was categorized into several status groups. For this purpose, a distribution-based classification into five equal groups (quintiles) was used, with the three middle groups being combined. This three-level scale, i.e. “low income” (1st quintile), “middle income” (2nd − 4th quintile) and “high income” (5th quintile), enables a comparison between the 20% of children and young people who grow up in the families with the best or worst financial situation and a broadly defined middle, which comprises 60% of children and young people [33]. In case of of incomplete families (e.g., divorced or single parents), the number of adults living in the household was included in the calculation of the financial situation. This means that for instance, in the case of single parents, only one person was included, and in the case of mixed families, other adults living in the household were included accordingly.

#### Mental health problems of children and adolescents

Mental health issues were evaluated using the internationally recognized and validated Strengths and Difficulties Questionnaire [34], which includes four problem scales: emotional problems, conduct problems, hyperactivity, and peer problems, each consisting of five items. Respondents rated each item on a three-point scale (0 = “not true” to 2 = “certainly true”). By summing the responses for all 20 items across these subscales, a total difficulties score was calculated, ranging from 0 to 40. Higher scores reflect more severe mental health problems. Additionally, established cut-off points [35] were applied to categorize participants into two groups: those with (“abnormal/borderline”, i.e. 13 points or higher) mental health problems and those without (“normal”, i.e. 0–12 points).

#### Personal, family and social factors

*Parental psychopathology* was assessed by parental depressive symptoms indicated by the eight-item Patient Health Questionnaire [36]. Each item was offered with four response options ranging from 1 = “not at all” to 4 = “nearly every day”. A sum score was calculated with values ranging from 0 to 24, whereby higher score indicates more depressive symptoms. Cronbach’s α ranged from 0.88 to 0.90 across measurement points.

The *Personal Resources Scale* by Bettge and Ravens-Sieberer [37] was administered to assess personal resources, such as problem-solving skills and optimism of children and adolescents. The items (e.g., “My child looks to the future with optimism/confidence”) were provided with four response options (1 = “not true” to 4 = “exactly true”). The whole scale comprises a total score transformed to values from 0 to 100. Higher scores point to more personal resources. Cronbach’s α ranged from 0.81 to 0.86 across measurement points.

Perceived *social support* was measured with four items of the Social Support Scale [38, 39]. The items (e.g., “How often has there been someone your child can count on to listen to when he/she needs to talk”) were answered using five response options (1 = “never” to 5 = “always”). The scale comprises a total value from 4 to 20. Higher scores indicate more perceived social support. Cronbach’s α ranged from 0.83 to 0.86 across measurement points. 

*Family cohesion* was assessed by four items from the Cohesion subscale of the Family Climate Scale (FCS, Schneewind, Beckmann [40]. The items (e.g., “In our family everybody cares about each other’s worries”) were answered on a four-point response scale (0 = “not true” to 3 = “exactly true”). A total sum score was calculated ranging from 0 to 16. A higher score indicates a better family cohesion. Cronbach’s α varied from 0.86 to 0.89 across measurement points.

### Statistical analysis

The change in mental health was examined by analyzing the percentage of abnormal scores on SDQ scales across all COPSY survey time points (T1–T7). These findings were compared with pre-pandemic BELLA data (T0), stratified by parental educational level (low, medium, high). Differences were tested using chi-square tests and effect sizes were calculated using Cramer’s V. To illustrate the courses of mental health problems over time, mean values of the SDQ subscales were calculated stratified by parental education and household income. Differences were tested using chi-square tests.

To test longitudinal changes in mental health (from T1 to T7) and to examine the association of socioeconomic status and risk/resource factors with mental health within (longitudinal) and between (cross-sectional) respondents, mixed model panel regression analyses were conducted. Coefficients were estimated representing a potential effect of each of the waves 2–7 (in comparison to wave 1), time-constant factors (age in 2023, gender, migration background, parental education, household income) and risk/resource factors (parental psychopathology, personal resources, family cohesion and social support). The simultaneous inclusion of these predictors in the multivariate models allows for a thorough controlling of confounding factors as well. In each model, a random intercept was included for every participant (to allow and represent scores differing individually). The random effects panel model was chosen, because it allows the simultaneous inclusion of time-constant and time-varying covariates.

The Relative Index of Inequality (RII) and Slope Index of Inequality (SII) for parental education (CASMIN) and household income was calculated for the most recent data (T7) following the linear regression approach described by Moreno-Betancur [41]. The Relative Index of Inequality (RII) measures relative inequality: a value of 1 means no inequality (the same risk), a value > 1 means higher risk for the disadvantaged group, and a value < 1 means lower risk for the disadvantaged group. The Slope Index of Inequality (SII) measures absolute inequality. It indicates the absolute difference in health status between the theoretically most disadvantaged and least disadvantaged groups: a value of 0 means no inequality, a positive/negative value means that the further away from 0, the greater the absolute inequality.

The panel regression analysis was performed using Stata 18, all other statistical analyses were computed with IBM SPSS 27.

## Results

### Sociodemographic

Pre-pandemic data was available for *n* = 1,580 families with children aged 7 to 19 years (M = 12.5; SD = 2.9; 52.3% female) who participated in the BELLA study. During and after the pandemic, a range of *n* = 1,586 (T1) until *n* = 1,701 (T5) families with children and adolescents aged 7 to 22 years (M = 14.4; SD = 4.6; 49.7% female) took part in at least one of the seven survey waves (T1-T7) of the COPSY study. The majority of parents had a medium level of education and approximately half of the families had a medium household income. Approximately one-fifth of the children and adolescents had a migration background. Further sociodemographic information is presented in Table 1.

**Table 1:** Sample characteristics

	BELLA Pre-Pandemic (2014–2017)		COPSY T1 Pandemic (May-Jun. 2020)		COPSY T2 Pandemic (Dec. 2020-Jan. 2021)		COPSY T3 Pandemic (Sep.-Oct. 2021)		COPSY T4 Pandemic (Feb. 2022)		COPSY T5 Pandemic (Sept.- Oct. 2022)		COPSY T6 Post-Pandemic (Oct.-Nov. 2023)		COPSY T7 Post-Pandemic (Oct. 2024)	
	*N* = 1,580		*N* = 1,586		*N* = 1,625		*N* = 1,618		*N* = 1,668		*N* = 1,701		*N* = 1,673		*N* = 1,505	
Age (mean SD)	12.5 (2.9)		12.3 (3.3)		12.7 (3.3)		13.3 (3.3)		12.9 (3.7)		13.1 (4,0)		13.5 (4.4)		13.1 (4.5)	
	**n**	**%**	**n**	**%**	**n**	**%**	**n**	**%**	**n**	**%**	**n**	**%**	**n**	**%**	n	%
Gender																
male	754	47.7	791	49.9	823	50.7	783	48.5	822	49.3	847	49.8	825	49.3	760	50.5
female	826	52.3	793	50.0	800	49.3	823	51.0	836	50.1	844	49.6	843	50.4	740	49.2
divers			1	0.1	1	0.1	9	0.6	9	0.5	9	0.5	5	0.3	5	0.3
Migration Background																
no	1379	87.8	1332	84.0	1355	84.4	1322	82.5	1374	83.1	1412	83.7	1383	83.3	1242	83.1
yes	193	12.2	254	16.0	251	15.6	280	17.5	279	16.9	275	16.3	278	16.7	252	16.9
Parental education																
low	349	22.1	260	16.4	267	16.4	251	15.5	266	15.9	234	13.8	216	12.9	193	12.8
medium	790	50.0	834	52.6	875	53.9	910	56.2	967	58.0	973	57.2	1003	60.0	913	60.8
high	411	26.0	460	29.0	453	27.9	433	26.8	409	24.5	461	27.1	418	25.0	369	24.5
n.a.	30	1.9	32	2.0	30	1.9	24	1.5	26	1.6	33	1.9	36	2.2	30	2.0
Household income																
low	303	19.2	248	15.6	261	18.0	289	17.9	259	15.5	246	14.5	242	14.5	250	16.6
medium	909	57.5	722	45.5	761	46.2	776	48.0	823	49.3	848	49.9	851	50.9	764	50.8
high	303	19.2	235	14.8	242	13.7	237	14.6	282	16.9	312	18.3	297	17.8	265	17.6
n.a.	65	4.1	381	24.0	361	22.2	316	19.5	304	18.2	295	17.3	283	16.9	226	15.0
Mental health problems																
norm./ borderline	1399	88.6	1303	82.2	1295	79.7	1329	82.1	1369	82.1	1445	85.0	1426	85.2	1290	85.7
abnormal	153	9.7	283	17.8	330	20.3	289	17.9	299	17.9	256	15.0	247	14.8	215	14.3

Absolute figures and percentage figures unweighted

There were statistically significant differences between the BELLA and the COPSY sample regarding migration background (*p*=.002; V = 0.05) and parental education (*p*<.001; V = 0.06). However, the actual effect-sizes were rather small.

### Socioeconomic status and mental health problems

To answer research question 1, descriptive analyses were calculated for each survey wave to examine the association between indicators of socioeconomic status (i.e. household income and parental education) and children’s and adolescents’ mental health problems. Preliminary analysis revealed medium correlation between parental education and household income (*r*=.42).

**Fig. 1:**
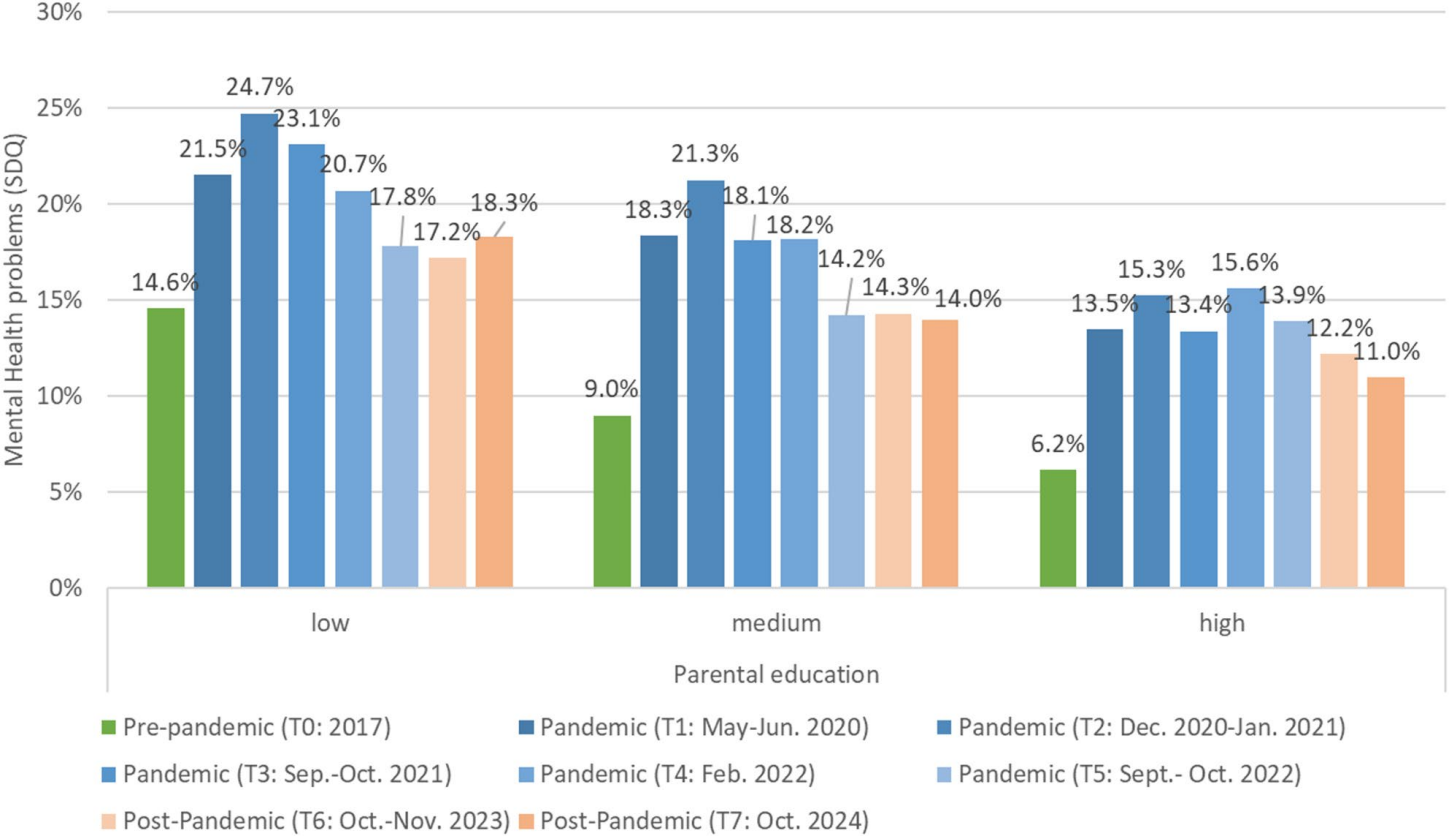
Prevalence of mental health problems (borderline/abnormal SDQ score) in children and adolescents by parental education. Note : weighted data, COPSY study T1-T7

Prevalence of mental health problems in children and adolescents by parental education are presented in Fig. 1 and Supplement Table 1. Thereby, a strong social gradient between children and adolescents from families with low parental education compared to peers from families with high parental education was observed prior to the COVID-19 pandemic (pre-pandemic: low 14.6% vs. medium 9.0% and high 6.2%, *p* < .001, V = 0.18). At the beginning of the pandemic an increase in mental health problems was observed across all status groups, whereby differences within the parental educational status groups were mitigated, but still significant (e.g. May-June 2020: low 21.5% vs. medium 18.3% and high 13.5%, *p*=.010, V = 0.08). During the COVID-19 pandemic prevalence of mental health problems of children and adolescents from families with low parental education increased substantially from 14.6% up to 24.7% in winter 2020/2021 and declined to 17.8% in autumn 2022. Similarly, but on a lower overall level, prevalence of mental health problems of children and adolescents from families with high parental education increased up to 15.6% in February 2022, declined to 13.9% in autumn 2022. After the COVID-19 pandemic, prevalence rates declined across all parental education status groups, but have not returned to pre-pandemic levels yet, i.e. across all groups 3.2% to 5.4% more children/adolescents have mental health problems than before the pandemic (post-pandemic: low 18.3% vs. medium 14.0% and high 11.0% *p* = .018, V = 0.07).

To answer research question 2, trends of mental health inequalities in children and adolescents from pre- until post-COVID-19 pandemic were assessed. Thereby, inequalities in mental health were observed for both SES-indicators, parental education and household income.

**Fig. 2:**
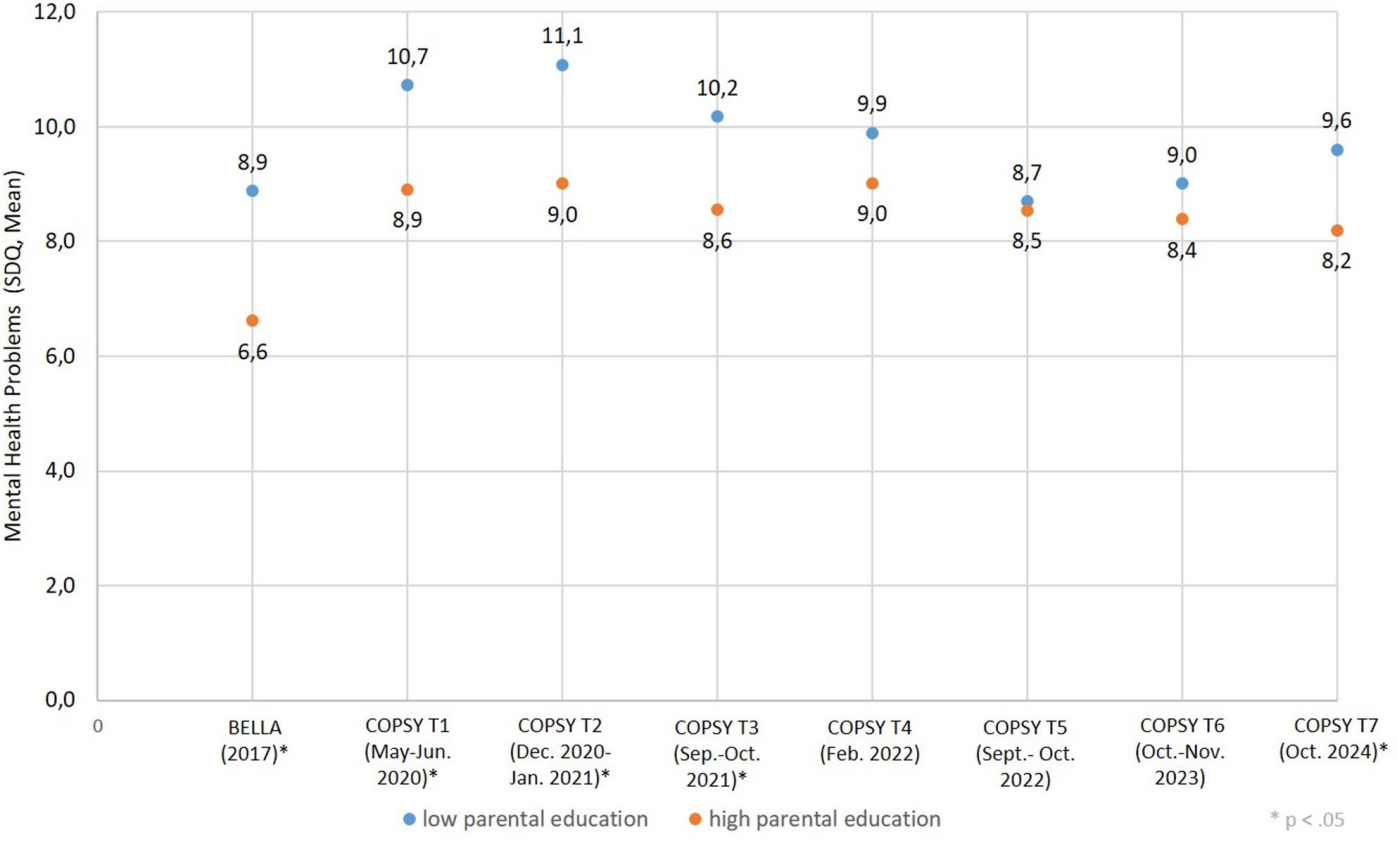
Mental Health inequalities by parental education

The development of mental health problems in children and adolescents with regard to their parents’ level of education is shown in Fig. 2 (further information see Supplement Table 2). Inequality in mental health was observed in that children and adolescents with a low level of parental education showed higher values for mental health problems pre-pandemic and during the first 1.5 years of the pandemic than their peers with a high level of parental education. However, this discrepancy narrowed towards the end of the pandemic and mean values in mental health problems were nearly the same for both status groups in autumn 2022. After the WHO officially declared the end of the COVID-19-pandemic (may 2023) in autumn 2023, inequalities in mental health problems have increased again, but not to the pre-pandemic level. Concerning parents’ level of education, the Relative Index of Inequality (RII) and Slope Index of Inequality (SII) for the most recent data were 1.16 and 1.26 respectively.

**Fig. 3:**
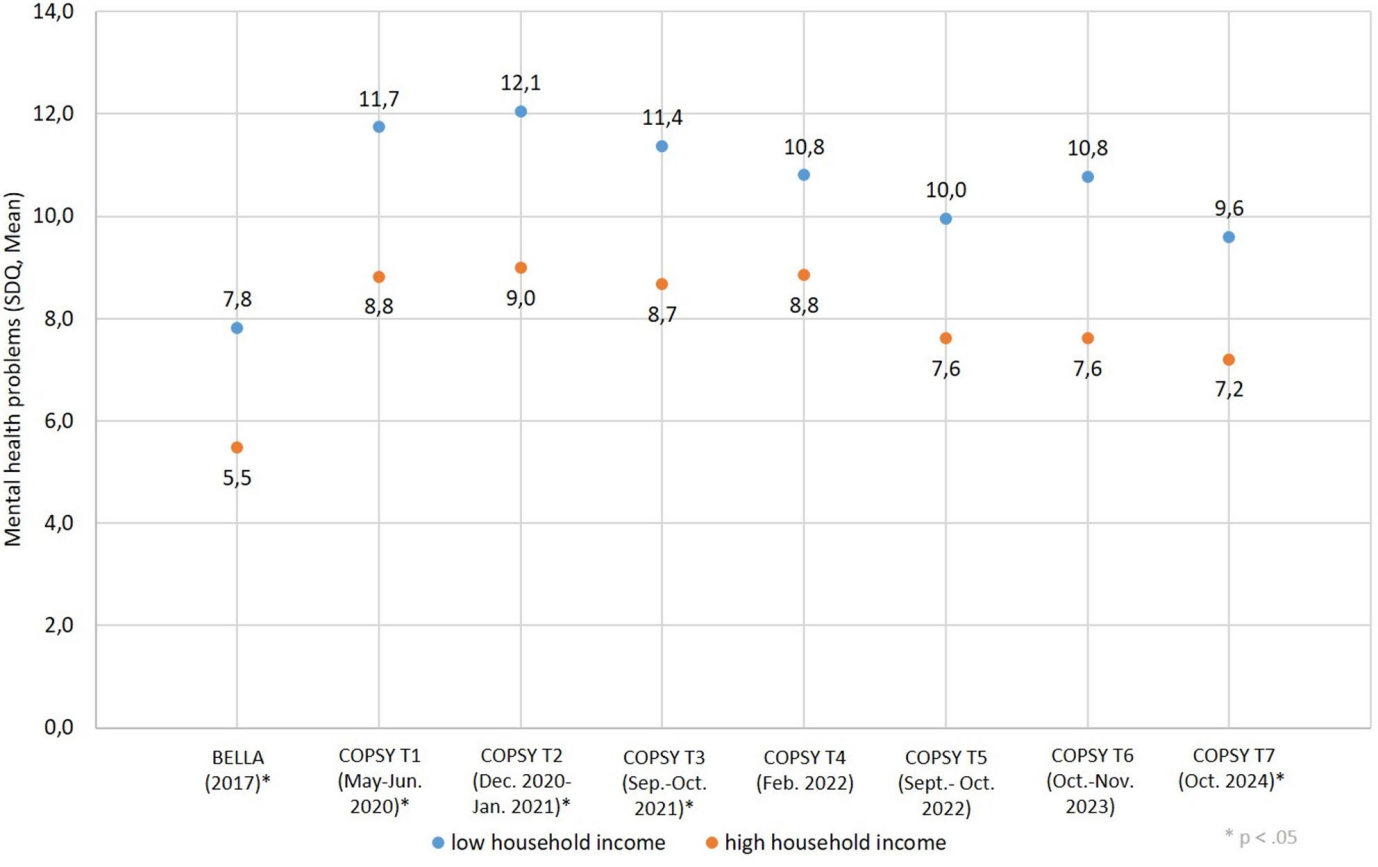
Mental Health inequalities by household income

The development of mental health problems in children and adolescents with regard to the household income is shown in Fig. 3 (further information see Supplement Table 3). At the beginning of the pandemic, there was also a high increase in the mean values for mental health problems among both children and adolescents with a low income and those with a high household income. During the course of the pandemic and also after the end, these inequalities in mental health problems persist. However, there was a temporary slight reduction in health inequalities in spring and autumn 2022. After the pandemic, the values for mental health problems are still higher than at the beginning of the pandemic, and the gap between children and adolescents with mental health problems appears to widen again in autumn 2023. With regard to the household income, the Relative Index of Inequality (RII) and Slope Index of Inequality (SII) for the most recent data were 1.32 and 2.42 respectively.

### Risk and resource factors for mental health problems

To answer research question 3, findings showed that during the first 1.5 years of the COVID-19-pandemic (T1-T4) parents with a low level of education reported significantly higher mean values of *depressive symptoms* than parents with a high level of education (e.g., T1: 6.4 vs. 4.4, *p*=.002, eta²=0.008). Towards the end of COVID-19-pandemic (T5), there were no differences in the depressive symptoms, whereas after the pandemic depressive symptoms increased in parents with low education to a higher extent compared to parents with a high education. Similar results indicated that parents with a low household income showed significantly higher mean values of *depressive symptoms* compared to parents with a high household income (e.g., T1: 7.3 vs. 4.4, *p*<.001, eta²=0.035) across all study measurement points, but with small effect sizes.

Heterogeneous results were found concerning resources for children’s and adolescent’s mental health. For instance, *social support* was during the pandemic (T1-T5) slightly better in families with low parental education or low household income than in families with high parental education or high household income (e.g., education: T5: 82.9 vs. 76.9, *p*<.001, eta²=0.014; income: T5: 81.6 vs. 79.3, *p* = .047, eta²=0.006). However, the association changed after the COVID-19-pandemic (T6-T7) and social support became better in families with a high household income compared to families with a low household income (T6: 81.0 vs. 78.2, *p*=.030, eta²=0.006). Moreover, families with low parental education reported a slightly better *family cohesion* compared to families with high parental education (e.g., T1: 78.2 vs. 71.2, *p*<.001, eta²=0.014). Families with low or high household income reported similar results concerning family cohesion during the COVID-19-pandemic (e.g., T2: 75.2 vs. 73.9, *p*=.314, eta²=0.003). In addition, children from families with a high household income had on average slightly better *personal resources* compared to peers from families with a low household income (e.g., T7: 74.8 vs. 70.1, *p*=.03, eta²=0.008). No significant differences were observed in personal resources in children and adolescents regarding the parental educational level. For all risk factors and resources small effect sizes were observed. For further information, see Supplement Tables 4 and 5.

Results of the panel regression model analysis are presented in Table 2. Findings revealed that female gender and higher age was associated with fewer mental health problems in children and adolescents, whereas no significant association was found for migration background. Low parental education was significantly associated with more mental health problems of children and adolescents. Likewise, but to a lesser extend, medium parental education as well as low household income was only statistically tendential associated with more mental health problems. Stronger personal, family, and social resources were linked to fewer mental health problems of children and adolescents, but the effect was small. The panel regression model explained 65.0% of the total variance in the mental health problems (SDQ scores, across persons and waves) and 26,4% of the variance in the mean SDQ-scores of the children and adolescents.

**Table 2:** Risk and resource factors of mental health problems in children and adolescents

Mental health problems (SDQ)	Coeffici-ent	std. err.	z	*P*>|z|	[95% conf. interval]	
Female	− .772	.287	-2.69	0.007	-1.334	− .209
Year of birth	.220	.047	4.63	0.000	.127	.313
Migration background	.047	.374	0.13	0.899	− .685	.779
Low parental education	1.267	.492	2.58	0.010	.306	2.234
Medium parental education	.616	.351	1.75	0.079	− .072	1.30
Low household income	.786	.465	1.69	0.091	− .126	1.699
Medium household income	.413	.354	1.17	0.243	− .280	1.106
Parental depressive symptoms	.286	.023	12.64	0.000	.330	
Personal resources	− .077	.006	-13.24	0.000	− .088	− .065
Family cohesion	− .027	.006	-4.82	0.000	− .039	− .016
Social support	− .039	.006	-6.75	0.000	− .050	− .028
T 2 (Dec. 2020-Jan. 2021)	− .039	.226	-1.50	0.133	− .782	.104
T 3 (Sep.-Oct. 2021)	− .647	.226	-2.86	0.004	-1.090	− .204
T 4 (Feb. 2022)	− .737	.223	-3.30	0.001	-1.175	− .300
T 5 (Sept.- Oct. 2022)	-1.184	.224	-5.30	0.000	-1.622	− .746
T 6 (Oct.-Nov. 2023)	-1.071	.224	-4.77	0.000	-1.511	− .632
T 7 (Oct. 2024)	-1.466	.228	-6.43	0.000	-1.913	-1.019
cons	9.694	.425	22.78	0.000	8.860	10.528

R2 across respondents and waves = 0.6498

R2 across respondents = 0.2639

## Discussion

The present large-scale population-based German studies (BELLA and COPSY) underline that children’s and adolescents’ mental health is significantly associated with the socioeconomic status (SES) of the family and adds important insights on the longitudinal development of socioeconomic inequalities in children’s and adolescents’ mental health comparing pre-pandemic with pandemic and post-pandemic data across almost seven years.

In line with previous research [1, 26], our study findings show a strong social gradient, which had already been observed prior to the COVID-19 pandemic in previous international studies, whereby children and adolescents from families with low parental education and low household income had 2–3 times higher levels of mental health problems compared to peers from families with a high parental education or high household income.

At the beginning of the COVID-19 pandemic in 2020, we observed an increase in mental health problems among children and adolescents across all socioeconomic status groups. Over the course of the pandemic, the social gradient in youth mental health shifted, including a slight increase in mental health inequalities related to household income at the beginning of the pandemic. Particularly towards the end of the pandemic, mental health inequalities narrowed, more noticeably in relation to parental educational background and to a lesser extent with regard to the level of the family’s household income. Similar results were reported by the UK Household Longitudinal Study that included 16,361 observations from 9,272 children measuring child mental health at ages 5 and 8 years between 2011 and 2021 [19]. Thereby, unexpectedly, children from traditionally advantaged groups (e.g. with employed parents, higher parental education and higher household income) saw larger declines in mental health than children from disadvantaged groups and child mental health has become more equal but at a worse overall level [19]. The authors explain the association by noting that although social disadvantage remains a risk factor, the pandemic acted as a ‘low-level equalizer’ by substantially worsening the mental health of previously privileged children. Differences in study results between Germany and the UK could be attributed to the fact that the United Kingdom (UK) has significantly higher social and economic inequality than Germany. While Germany ranks stably in the middle of the field in a European comparison, the UK is one of the countries with the highest income inequality in Europe.

Moreover, previous analyses of the COPSY study revealed that low parental education was correlated with higher initial mental health problems in children and adolescents at the beginning of the pandemic in 2020, but was not significantly associated with increasing mental health problems during the pandemic until autumn 2022 [42]. On the one hand, it can be assumed that social isolation and limited access to services during the COVID-19 pandemic has brought unfavourable experiences closer to historically advantaged groups [19]. Parents experienced less childcare support due to closed schools and kindergarten and less social support by other family members (e.g., grandparents) or professional carer (e.g., nannies) due to social distancing regulation. On the other hand, economic support measures in Germany during the pandemic (e.g., a child bonus for each family as well as higher social welfare benefits) may have brought some relief to socioeconomically disadvantaged families. In comparison to the mostly completed level of parental education in this stage of life, household income can be subject to major changes, particularly in times of crises and families with one or more children.

In addition, there might be potential developmental and age-dependent effects. For example, the transition of adolescents to university is often considered as a critical period, whereby parental socioeconomic status remains a significant predictor of mental health but often acts through different mediators, such as impulsivity and social support, rather than direct parental supervision [43]. Another perspective argues that mental health problems may become more prevalent as children grow older due to increased social comparisons, which makes them more sensitive to their family’s relative position in the social hierarchy [44]. This suggests that the broad age range in our sample may have influenced the observed associations, and future research should examine whether these effects differ across developmental stages.

Concerning the second research question and to the best of our knowledge, there are no studies to date available that have analysed the longitudinal development of mental health inequalities two years after the end of the COVID-19 pandemic. Our study findings show that after the pandemic, inequalities in children’s and adolescents’ mental health increased again, but at an overall higher level for all status groups compared to the pre-pandemic period. Therewith, our study results confirm our first hypothesis that low parental SES is associated with higher mental health problems in children and adolescence before, during, and after the COVID-19-pandemic. In terms of the second research hypothesis that mental health inequalities between children with a low SES compared to children with a high SES increased during the COVID-19 pandemic, findings showed a complex pattern. In order to be able to interpret these results, it is worth taking a deeper look into the research field of health inequality: Previous studies have shown that families with a low SES are exposed to a higher risk of accumulation of risk factor [45, 46], not only in families where children experience poverty at an early age [47], but also in low income neighbourhood, which is associated with a higher risk of mental health problems in childhood [48]. Results from the German BELLA study with 2,111 participants aged 7–17 years demonstrated that during periods of accumulated individual stressful life situations, families with high parental education showed greater resilience and fewer mental problems in their children [49]. Nowadays, multiple global crises such as the COVID-19 pandemic, the war in Ukraine, economic instabilities and security policy changes, do not only accumulate at the individual level, but also at the social level. As part of the growing body of research on well-being and mental health during global crises, our study suggests that even parental education, typically seen as a protective factor, may not be sufficient to mitigate the long-lasting strain caused by high levels of individual and societal stress. We also observed a strong increase in mental health problems in children and adolescents with highly educated parents, which might be also a result of social isolation and social distancing during the pandemic, which affected all children regardless their socioeconomic background.

According to previous research, high levels of parental mental health problems, parental stress, parental strain, and low parental competence are associated with poorer child behavioural and emotional outcomes, especially in low income and socioeconomic disadvantaged families [50, 51]. Results from a German panel study with 1,771 participants, conducted between March 2020 and April 2021, showed that the shift of childcare and schooling responsibility from formal institutions to private households during the pandemic placed immense stress on families. Parents with younger children, lower incomes, and those working from home experienced particularly high levels of psychological distress during this time [52]. Another German representative survey of 258 paediatricians documented the development of 7,818 infants and toddlers, whereby around a fifth of all parents (21.5%) reported moderate to clinically significant psychological stress in 2022 (compared to 15.7% in 2015). Parents with psychological distress were significantly more likely to express doubts about their own parenting skills than parents without psychological distress [53]. In addition to this research, our study results show that parents with a low level of education and low household income are more likely to report depressive symptoms. In accordance to the family stress model [54], parental mental health and parent relationship quality might contribute to the association between family socioeconomic conditions and children’s mental health outcomes.

A notable finding of our study is that families with a low level of education were surprisingly slightly better equipped with resources such as a good family cohesion and good social support, especially during the pandemic than families with a high level of parental education. Despite these internal strengths and the provision of government financial aid, mental health inequalities remained or widened again after the pandemic. According to our results, a higher risk of mental health problems for the disadvantaged group (RII) as well as the absolute difference in mental health status (SII) were more pronounced for young people from low-income families compared to families with low parental education. The pandemic has exacerbated existing social inequalities, necessitating a wide range of measures to support socially disadvantaged families. Although many support measures were introduced, there was also criticism of their insufficient reach and the bureaucratic hurdles that placed an additional burden on low-income households in particular.

Moreover, we assume that pandemic measures such as social distancing has affected families across all socioeconomic status groups, including academic families, who might live further away from their parents or grandparents. The overserved, but low effect of resources such as social support, family cohesion, and personal resources was also visible in the panel regression analysis. We assume that family socioeconomic status is strongly associated with the mental health of children and adolescents, but should not be considered isolated from other determinants such as personal or social resources. For instance, a study with a global sample of 73,182 children aged 10 and 12 years from 25 countries/regions found that the family-child relationship is the strongest mediator between material deprivation and subjective well-being [55]. In turn, an improvement of the quality of social relationships among economically less privileged children is recommended in order to reduce negative effects of material deprivation on child well-being [55]. The observed associations are complex, whereby the subjective assessments of social support and family cohesion might also play a role. Nonetheless, crises also seem to have the potential to mobilize resources - even, and especially, in families with low socioeconomic status.

Further, our study results confirm the third hypothesis that risk or resource factors (e.g., parental psychopathology, personal resources, family cohesion, social support) are associated to children’s and adolescents’ mental health. In summary, the factors analysed relating to family socioeconomic status as well as risk and resource factors explain almost two thirds of the variance in mental health problems among children and adolescents over time. This indicates the great importance of these factors in enabling children and adolescents to grow up healthily. Therewith, our research underlines previous approaches that pathways to inequalities in health might be complex and inter-related. However, they are largely influenced by variations in the distribution of power and resources that shape the economic, material, and psychosocial environments in which children are raised [21].

### Strengths and limitations

To our knowledge, this study is one of the first large-scale, population-based longitudinal studies that combines pre-pandemic data from the BELLA study with data from the ongoing COPSY study. The COPSY study began at the onset of the pandemic and has since assessed mental health trends throughout the pandemic as well as in the post-pandemic period, now spanning almost seven years. This unique approach contributes robust and comparable findings to the field of social inequality in child and adolescent mental health in Germany. Strengths of the BELLA/COPSY study are their comparable, well conceptualized and methodologically sound and stringent design and the application of well established, validated questionnaires, which allow for systematic international comparisons.

The limitations of this study are that mental health problems were parent-proxy reported, which may differ from self-reports by the youth themselves. This approach was chosen to ensure consistency across the longitudinal assessment waves and to minimize participant burden, as the study was originally not designed for such a long follow-up period. The studied relationships are associations and do not prove a causal link between the examined constructs. Also, findings may not be generalizable to other countries. Additionally, like all studies, we focused on specific risk and protective factors, which may have led to the exclusion of other relevant factors. There are some small but statistically significant differences between the BELLA and the COPSY sample regarding sociodemographic variables. But the effect-sizes are rather small and their impact on the association between socioeconomic factors and health outcomes might be neglectable.

### Implications for future research

Especially in substantial crisis situations such as the COVID-19 pandemic, differences concerning the examined indicators of socioeconomic status became evident. For future research, it would be advisable not only to assess the objective indicators of socioeconomic status of the parents, but also to take greater account of the individual assessment of children’s and adolescents’ socioeconomic position. Recent study findings indicate that adolescents’ reports of subjective socioeconomic status (e.g., their opinion about the family financial situation) and subjective social status (i.e., their perceived social status in comparison to others) predicted self-reported measures of mental health to the same degree or even better than parent reports of both subjective and objective SES [56]. The self-reporting by adolescents on their subjectively perceived socioeconomic status can therefore be a suitable instrument, not only to have a relative measure of their own position in society, but also to perspectival map trends in health inequality.

Moreover, future research on inequalities in mental health should also consider the multidimensionality of childhood socioeconomic deprivation. Recent research highlights that early childhood deprivation has a dynamic and complex structure that must be considered when studying the early years period [57]. In addition to the ‘classic’ indicators such as parental education and income, it is also worth including socio-spatial indicators such as place of residence and access to educational institutions in order to improve equal (mental) health opportunities of children and adolescents. Our results further reveal crisis-sensitive changes in socioeconomic inequalities, affecting even previously privileged groups and compromising their health. Despite these shifts, inequalities in children’s and adolescents’ mental health continue to persist.

## Conclusions

In summary, our study underlines that socioeconomic status has a decisive influence on the mental health of children and adolescents. Surprisingly, equalities in mental health of the youth did not increase during the pandemic, but persisted. In 2022, there was even a temporary decrease in the youth’ mental health inequality gap between high vs. low parental educational groups visible. Resource factors (i.e., personal resources, family cohesion, social support) had a small but significant positive effect on mental health in children and adolescents.

Our findings underscore the importance of targeted health promotion and prevention programs to support children and adolescents’ well-being in turbulent times. Political, educational, and health programs that address social and health inequalities can play a crucial role in improving children’s mental health. In Germany, a whole range of political and health-related measures already exists to improve the (mental) health of young people. For instance, recent policy initiatives such as an interministerial working group established at the beginning of 2023, addresses child and adolescent mental health inequalities by developing cross-sectoral recommendations for child health promotion across different life stages, childcare settings, schools, and health care. These health promotion and prevention efforts address both: individual and societal levels. The long-term effectiveness of such initiatives depends on their continuous and nationwide implementation rather than short-term or pilot-based funding structures. In particular, evidence-informed school-based mental health promotion approaches such as low-threshold mental health coaches or social workers, should be scaled up nationwide. In addition, community-based approaches, including municipal prevention chains and family-oriented services embedded, aim to strengthen family resources and social support across different stages of childhood and adolescence, e.g., family midwives in Germany.

However, these holistic approaches are not yet implemented nationwide due to the federal structure of the country. Based on our findings, future policy efforts should prioritize the expansion of low-threshold, integrated prevention services in schools and neighbourhoods, with resource allocation guided by social disadvantage indicators.

## Supplementary Information

Supplementary Material 1.

## Data Availability

The data that support the findings of this study are available from the corresponding author upon reasonable request.

## Abbreviations

BELLA: Behaviour and Well-being of Children and Adolescents in Germany study
COPSY: Covid-19 and Psychological Health study
SDQ: Strengths and Difficulties Questionnaire
