# Supplementary material for: Tracing socioeconomic inequalities in children’s and adolescent’s mental health: longitudinal study findings from 2017 to 2024
Source: BMC Public Health. 2026 Feb 17;26:711. doi: 10.1186/s12889-026-26577-0 (PMC12930680; doi:10.1186/s12889-026-26577-0)
Supplement: Supplementary file 1 — Supplementary Material 1. [file 12889_2026_26577_MOESM1_ESM.docx]

**Supplementary material**

**Supp. Table 1.** Prevalence of mental health problems (Percentage of abnormal/borderline SDQ total scores) by parental education.

| **Survey time point** | **Prevalence of mental health problems**  **(% of abnormal/borderline**  **total SDQ score)** | | | ***p*** | **V** |
| --- | --- | --- | --- | --- | --- |
|  | Low  Parental education | Medium Parental education | High Parental education |  |  |
| Pre-pandemic (BELLA) | 14.6% | 9.0% | 6.2% | **<.001** | 0.18 |
| Pandemic (COPSY T1: May-Jun. 2020) | 21.5% | 18.3% | 13.5% | **.010** | 0.08 |
| Pandemic (COPSY T2: Dec. 2020-Jan. 2021) | 24.7% | 21.3% | 15.3% | **.004** | 0.08 |
| Pandemic (COPSY T3: Sep.-Oct. 2021) | 23.1% | 18.1% | 13.4% | **.005** | 0.08 |
| Pandemic COPSY (T4: Feb. 2022) | 20.7% | 18.2% | 15.6% | .270 | 0.04 |
| Pandemic (COPSY T5: Sept.- Oct. 2022) | 17.8% | 14.2% | 13.9% | .301 | 0.04 |
| Post-Pandemic (COPSY T6: Oct.-Nov. 2023) | 17.2% | 14.3% | 12.2% | .176 | 0.05 |
| Post-Pandemic (COPSY T7: Oct. 2024) | 18.3% | 14.0% | 11.0% | .**018** | 0.07 |

*Note:* p: Chi²; V: Cramers’s V

**Supp. Table 2.** Mean scores in mental health problems of children and adolescents and parental education from 2017 until 2024

| **Mental health problems**  **Mean SDQ total Score** | **BELLA Study** | **COPSY T1** | **COPSY T2** | **COPSY T3** | **COPSY T4** | **COPSY T5** | **COPSY T6** | **COPSY T7** |
| --- | --- | --- | --- | --- | --- | --- | --- | --- |
|  | (2017) | (May-Jun. 2020) | (Dec. 2020-Jan. 2021) | (Sep.-Oct. 2021) | (Feb. 2022) | (Sept.- Oct. 2022) | (Oct.-Nov. 2023) | (Oct. 2024) |
| Low parental education  (SD; 95% CI) | 8.9  (6.11; 8.24-9.52) | 10.7  (6.23; 9.97-11.49) | 11.1  (7.11; 10.22- 11.94) | 10.2  (7.00; 9.31-11.05) | 9.9  (6.68; 9.03- 10.76) | 8.7  (6.84; 7.84- 9.55) | 9.0  (6.93; 8.11- 9.90) | 9.6  (6.70; 8.71 – 10.57) |
| Medium parental education  (SD; 95% CI) | 7.6  (5.2; 7.24-7.96) | 10.4  (5.99; 9.94-10.76) | 10.3  (6.50; 9.86- 10.72) | 9.8  (6.45;- 9.41-10.25) | 9.8  (6.27; 9.40- 10.22) | 8.7  (5.98; 8.28- 9.07) | 9.0  (5.96; 8.60- 9.39) | 8.6  (6.09; 8.21 – 9.07) |
| High parental education  (SD; 95% CI) | 6.6  (6.6; 6.13-7.12) | 8.9  (5.71; 8.39- 9.43) | 9.0  (5.98; 8.46- 9.56) | 8.6  (6.35;- 7.96- 9.16) | 9.0  (6.46; 8.45- 9.59) | 8.5  (5.88; 8.04- 9.04 | 8.4  (5.90; 7.88- 8.89) | 8.2  (5.70; 7.66 – 8.66) |
| p-value | **0.000** | **<.001** | **<.001** | **<.001** | 0.06 | 0.913 | 0.165 | **0.014** |
| F | 16.515 | 11.097 | 9.871 | 7.023 | 2.814 | 0.091 | 1.802 | 4.302 |

* *Note*: SDQ: Strengths and Difficulties Questionnaire; SD: Standard Deviation; CI: Confidence Interval

**Supp. Table 3.** Mean scores in mental health problems of children and adolescents and household income from 2017 until 2024

| **Mental health problems**  **Mean SDQ total Score** | **BELLA Study** | **COPSY T1** | **COPSY T2** | **COPSY T3** | **COPSY T4** | **COPSY T5** | **COPSY T6** | **COPSY T7** |
| --- | --- | --- | --- | --- | --- | --- | --- | --- |
|  | (2017) | (May-Jun. 2020) | (Dec. 2020-Jan. 2021) | (Sep.-Oct. 2021) | (Feb. 2022) | (Sept.- Oct. 2022) | (Oct.-Nov. 2023) | (Oct. 2024) |
| Low household income  (SD; 95% CI) | 7.8  (5.52; 7.29-8.34) | 11.7  (6.14; 10.97- 12.50) | 12.1  (6.77; 11.22- 12.88) | 11.4  (7.13; 10.54- 12.21) | 10.8  (6.52; 9.99- 11.62) | 10.0  (6.57; 9.11- 10.81) | 10.8  (6.90; 9.90- 11.66) | 9.6  (6.68; 8.70 – 10.46) |
| Medium household income  (SD; 95% CI) | 8.0  (5.63; 7.65- 8.40) | 10.4  (6.08; 9.93- 10.83 | 10.4  (6.74; 9.91- 10.88) | 9.7  (6.65; 9.22- 10.17) | 9.9  (6.63; 9.40- 10.32) | 8.8  (6.19; 8.42- 9.26) | 8.9  (6.01; 8.46- 9.28) | 9.0  (6.17; 8.57 – 9.45) |
| High household income  (SD; 95% CI) | 5.5  (3.67; 4.97- 5.98) | 8.8  (5.31; 8.15- 9.49) | 9.0  (5.71; 8.29- 9.70) | 8.7  (5.86; 7.95- 10.22 | 8.8  (5.56; 8.22- 9.48) | 7.6  (5.27; 7.04- 8.19) | 7.6  (5.56; 6.99- 8.23) | 7.2  5.23; 6.63 – 7.86) |
| p-value | **0.000** | **0.000** | **0.000** | **0.000** | **0.002** | **0.000** | **0.000** | **0.000** |
| F | 19.139 | 14.837 | 13.944 | 11.702 | 6.485 | 10.360 | 18.564 | 11.306 |

* *Note*: SDQ: Strengths and Difficulties Questionnaire; SD: Standard Deviation; CI: Confidence Interval

**Supp. Table 4.** Mean scores for risk and resource factors by parental education

|  | **COPSY T1** | **COPSY T2** | **COPSY T3** | **COPSY T4** | **COPSY T5** | **COPSY T6** | **COPSY T7** |
| --- | --- | --- | --- | --- | --- | --- | --- |
|  | (May-Jun. 2020) | (Dec. 2020-Jan. 2021) | (Sep.-Oct. 2021) | (Feb. 2022) | (Sept.- Oct. 2022) | (Oct.-Nov. 2023) | (Oct. 2024) |
| **Resources (mean values)** |  |  |  |  |  |  |  |
| *Personal resources (0-100)* |  |  |  |  |  |  |  |
| low parental education | 69.07 | 67.69 | 68.26 | 66.72 | 71.74 | 72.76 | 69.73 |
| high parental education | 70.06 | 68.38 | 68.28 | 68.16 | 67.84 | 70.81 | 70.16 |
| p-value | .358 | .109 | .520 | .428 | .067 | .347 | .929 |
| Effect size | 0.002 | 0.004 | 0.001 | 0.002 | 0.005 | 0.002 | <0.001 |
| *Family cohesion (0-100)* |  |  |  |  |  |  |  |
| low parental education | 78.22 | 80.00 | 78.75 | 79.45 | 80.63 | 80.35 | 82.49 |
| high parental education | 71.23 | 72.50 | 72.58 | 73.66 | 73.23 | 74.33 | 74.28 |
| p-value | **<.001** | **<.001** | **<.001** | **.002** | **<.001** | **<.001** | **<.001** |
| Effect size | 0.014 | 0.022 | 0.012 | 0.011 | 0.019 | 0.012 | 0.025 |
| *Social support (0-100)* |  |  |  |  |  |  |  |
| low parental education | 80.80 | 80.40 | 81.80 | 80.80 | 82.89 | 82.39 | 83.18 |
| high parental education | 74.79 | 73.79 | 75.82 | 74.41 | 76.90 | 77.99 | 77.85 |
| p-value | **.001** | **<.001** | **<.001** | **<.001** | **<.001** | **.008** | **.004** |
| Effect size | 0.013 | 0.016 | 0.012 | 0.015 | 0.014 | 0.008 | 0.011 |
| **Risk factor (mean values)** |  |  |  |  |  |  |  |
| *Parental depressive symptoms (0-24)* | |  |  |  |  |  |  |
| low parental education | 6.44 | 6.97 | 6.03 | 5.93 | 5.21 | 5.88 | 6.86 |
| high parental education | 4.44 | 5.65 | 5.29 | 5.79 | 5.22 | 5.10 | 5.45 |
| p-value | **.002** | **.027** | .079 | .352 | .149 | .105 | **.001** |
| Effect size | 0.008 | 0.006 | 0.003 | 0.001 | 0.002 | 0.003 | 0.009 |

*Note:* effect size = eta-square

**Supp. Table 5.** Mean scores for risk and resource factors by household income

|  | **COPSY T1** | **COPSY T2** | **COPSY T3** | **COPSY T4** | **COPSY T5** | **COPSY T6** | **COPSY T7** |
| --- | --- | --- | --- | --- | --- | --- | --- |
|  | (May-Jun. 2020) | (Dec. 2020-Jan. 2021) | (Sep.-Oct. 2021) | (Feb. 2022) | (Sept.- Oct. 2022) | (Oct.-Nov. 2023) | (Oct. 2024) |
| **Resources (mean values)** |  |  |  |  |  |  |  |
| *Personal resources (0-100)* |  |  |  |  |  |  |  |
| low household income | 67.97 | 62.94 | 63.49 | 67.40 | 67.61 | 66.68 | 70.14 |
| high household income | 69.21 | 69.53 | 71.12 | 69.89 | 69.19 | 73.26 | 74.76 |
| p-value | .681 | .006 | **<.001** | **.004** | .508 | **<.001** | **<.001** |
| Effect size | 0.001 | 0.013 | 0.018 | 0.012 | 0.001 | 0.015 | 0.017 |
| *Family cohesion (0-100)* |  |  |  |  |  |  |  |
| low household income | 75.16 | 75.03 | 63.49 | 77.20 | 75.52 | 75.60 | 77.03 |
| high household income | 73.93 | 75.63 | 74.48 | 76.41 | 75.94 | 76.98 | 75.41 |
| p-value | .450 | **.038** | .676 | **.015** | .745 | .390 | .589 |
| Effect size | 0.002 | 0.008 | 0.001 | 0.009 | .001 | 0.002 | 0.001 |
| *Social support (0-100)* |  |  |  |  |  |  |  |
| low household income | 77.10 | 77.08 | 77.98 | 79.12 | 81.64 | 78.15 | 80.92 |
| high household income | 74.05 | 74.99 | 77.73 | 75.04 | 79.28 | 81.04 | 81.74 |
| p-value | .314 | .113 | .333 | **.003** | **.047** | **.047** | **.030** |
| Effect size | 0.003 | 0.005 | 0.002 | 0.013 | .007 | 0.006 | 0.008 |
| **Risk factor (mean values)** |  |  |  |  |  |  |  |
| *Parental depressive symptoms (0-24)* | |  |  |  |  |  |  |
| low household income | 7.32 | 8.30 | 7.04 | 7.12 | 6.54 | 6.69 | 7.25 |
| high household income | 4.44 | 5.24 | 5.34 | 4.99 | 4.57 | 4.37 | 4.57 |
| p-value | **<.001** | **<.001** | **<.001** | **<.001** | **<.001** | **<.001** | **<.001** |
| Effect size | 0.035 | 0.024 | 0.013 | 0.020 | .016 | 0.023 | 0.032 |

*Note:* effect size = eta-square
